# Protective effect of *Eucommia ulmoides* Oliver male flowers on ethanol‐induced DNA damage in mouse cerebellum and cerebral cortex

**DOI:** 10.1002/fsn3.2882

**Published:** 2022-04-22

**Authors:** Yanxia Ding, Yantong Wu, Juan Chen, Zhaoli Zhou, Bing Zhao, Rihong Zhao, Yuzi Cui, Qin Li, Yue Cong

**Affiliations:** ^1^ 12411 Institute of Pharmacy Engineering Center of Henan Province Eucommia ulmoides Cultivation and Utilization School of Pharmacy Henan University Kaifeng China; ^2^ 12411 State Key Laboratory of Crop Stress Adaptation and Improvement Henan Joint International Laboratory for Crop Multi‐Omics Research School of Life Sciences Henan University Kaifeng China

**Keywords:** cerebellum, cerebral cortex, DNA damage, ethanol, *Eucommia ulmoides* Oliver male flower, oxidative stress

## Abstract

Ethanol is a principal ingredient of alcoholic beverages with potential neurotoxicity and genotoxicity, and the ethanol‐associated oxidative DNA damage in the central nervous system is well documented. Natural product may offer new options to protect the brain against ethanol‐induced neurotoxicity. The male flower of *Eucommia ulmoides* (EUF) Oliver has been extensively utilized as the tea, the healthy hot drink on the market. In this study, 19 constituents in the effective fraction of EUF were identified by ultra‐performance liquid chromatography–tandem mass spectrometry (UPLC–MS/MS). In the single‐cell gel electrophoresis assay, EUF was observed to ameliorate DNA damage in mouse cerebellum and cerebral cortex caused by acute ethanol administration, which was further confirmed by the morphological observation. The protective effects of EUF were associated with increasing total superoxide dismutase (T‐SOD) and glutathione peroxidase (GSH‐PX) activities, and a decrease in nitric oxide (NO), malondialdehyde (MDA), 8‐hydroxy‐2′‐deoxyguanosine (8‐OHdG), and kelch‐like ECH‐associated protein‐1 (Keap1) levels. Molecular docking results demonstrated that compounds **4**, **7**, **9**, and **16** from EUF have a strong affinity to the Keap1 Kelch domain to hinder the interaction of nuclear factor‐erythroid 2‐related factor 2 (Nrf2) with Keap1. These findings suggest that EUF is a potent inhibitor of ethanol‐induced brain injury possibly via the inhibition of oxidative stress.

## Graphical abstract

Nineteen constituents in the effective fraction of the *Eucommia ulmoides* male flower (EUF) were identified by ultra‐performance liquid chromatography–tandem mass spectrometry (UPLC–MS/MS). The effective fraction of EUF decreases DNA damage in mouse brain caused by ethanol administration, which was associated with increasing total superoxide dismutase (T‐SOD) and glutathione peroxidase (GSH‐PX) activities, and a decrease in nitric oxide (NO), malondialdehyde (MDA), 8‐hydroxy‐2′‐deoxyguanosine (8‐OHdG), and kelch‐like ECH‐associated protein‐1 (Keap1) levels.

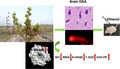

## 1 INTRODUCTION

Alcohol abuse has become one of the serious problems that harm human health. It can cause brain damage, cognitive impairment, loss of motor coordination function, and corresponding changes in psychological function (Jonas et al., 2000). Therefore, the central nervous system is the main target of alcohol, which leads to the genotoxicity of the central nervous system. At present, it has been proved that acute and long‐term use of alcohol can result in different degrees of oxidative damage to DNA in different brain regions, and then generate pathological changes such as necrosis, apoptosis, and inflammation of brain nerve cells, thus leading to various brain diseases. (Harper & Matsumoto, 2005) In recent years, some nature products were proved for having a protective effect on oxidative DNA damage elicited by alcohol.

*Eucommia ulmoides* Oliv., as a Chinese medicinal homologous food, is a unique and precious medicinal plant occurring in China. The bark, cortex Eucommiae, together with its leaf, folium Eucommiae, has been listed in “China Pharmacopoeia” (2020 Edition). As a deciduous and dioecious tree, the proportion between the male and female trees is equal in natural distribution. The male flowers, consisting of 5–12 linear stamens without perianth, are crowded in the bract in young shoots and developed into a popular health tea on the market. It has rich biologically active compounds, such as phenylpropanoids, iridoids, flavonoids, amino acids, vitamins as well as minerals (Xing et al., 2019). These bioactive compounds possess many pharmacological functions, including antioxidative stress, antiaging, sedation and hypnosis, anti‐inflammation, analgesia, bacteriostat, and immune modulation (Liu et al., 2020). However, our understanding of its neuroprotective effect still remains limited.

In this study, the acute brain nerve DNA damage model in mice was induced by the intraperitoneal (ip) injection of alcohol. The protective effect of EUF on alcohol‐induced DNA damage in mouse brain tissue was studied by the comet assay and brain tissue section observation. On this basis, this work further detected the effects on the physiological indexes related to oxidative damage in cerebellum and cerebral cortex of mice to reveal the protective effect of EUF on DNA damage in brain tissue induced by alcohol. Since *Eucommia ulmoides* Oliv. cultivation in China has a wide geographical distribution, this study will be helpful to promote its comprehensive development and utilization.

## 2 MATERIALS AND METHODS

### 2.1 Materials

*Eucommia ulmoides* male flower (EUF) were obtained from Lingbao Dehui Ecological Technology Co., Ltd (Henan, China) and authenticated by Associate Prof. Yue Cong at Henan University. A voucher specimen (2,017,007) was deposited at the Engineering Center of Henan Province *Eucommia ulmoides* Cultivation and Utilization, Henan University, China. A total of 200 male Kunming mice, weighting 22 g ± 2, were obtained from the Experimental Animal Center of Zhengzhou University, China. The animals were handled humanely in accordance with the guidelines of the National Research Council's (USA) document “NIH Guide for the Care and Use of Laboratory Animals.” Before the protocol was implemented, it was approved first by the Animal Care and Use Committee of Henan University.

### 2.2 Chemical and reagents

Deionized water was prepared using a Milli‐Q reagent water system (Millipore). Acetonitrile (high‐performance liquid chromatography (HPLC) grade) was obtained from Merck. Formic acid of HPLC grade was purchased from Fisher. Assay kits for NO, MDA, SOD, and GSH‐PX were products of the Nanjing Jiancheng Bioengineering Institute (China). 8‐OHdG was provided by Shanghai Westang Bio‐Tech. Co. Ltd. The reference substances of **1**, **3**, **4**, **5**, **8**, **9**, **12**, and **18** were purchased from the Chinese National Institute for the Control of Pharmaceutical and Biologiceal Products (Beijing, China). All other organic and inorganic reagents (analytical grade) were provided by local or international suppliers.

### 2.3 Preparation and UPLC–HRMS/MS analysis of sample A

The dried EUF (200 g) were ground to a fine powder and then extracted with a solution containing 50% (v/v) ethanol in water using a JHBE‐100A homogenization extractor (Zhijing Biotechnology Co.) three times, 4 min each time. The 50% ethanol extract was filtered, and the filtrate was concentrated under reduced pressure to a residue. The residue was mounted on a macroporous resin D101 column chromatography and eluted successively with EtOH–H_2_O (0:10; 3:7; 6:4) to collect the 60% EtOH elution and concentrated under reduced pressure to gain sample A (2.87 g).

UPLC–MS/MS analysis was conducted on the Q Exactive Plus mass spectrometry (Thermo Fisher Scientific). The UPLC was carried out on the vanquish‐flex with a Hypersil GOLD column (2.1 × 100 mm.1.9 um). The column temperature was set to 30°C and 1 μl of the sample was loaded. The mobile phase A was HPLC grade H_2_O with 0.1% (v/v) formic acid and phase B used was HPLC grade acetonitrile. The gradient elution conditions were set as follows: From 0 to 2 min, the mobile phase B increases to 10%; from 2 to 10 min, the mobile phase B increases to 50%; from 10 to 10.1 min, the mobile phase B increases to 80%; from 10.1 to 13 min, the mobile phase B was kept at 80%; from 13 to 14 min, the mobile phase B increases to 95%; from 14 to 18 min, the mobile phase B decreased to 10%. The flow rate was 0.3 ml/min.

**FIGURE 1:**
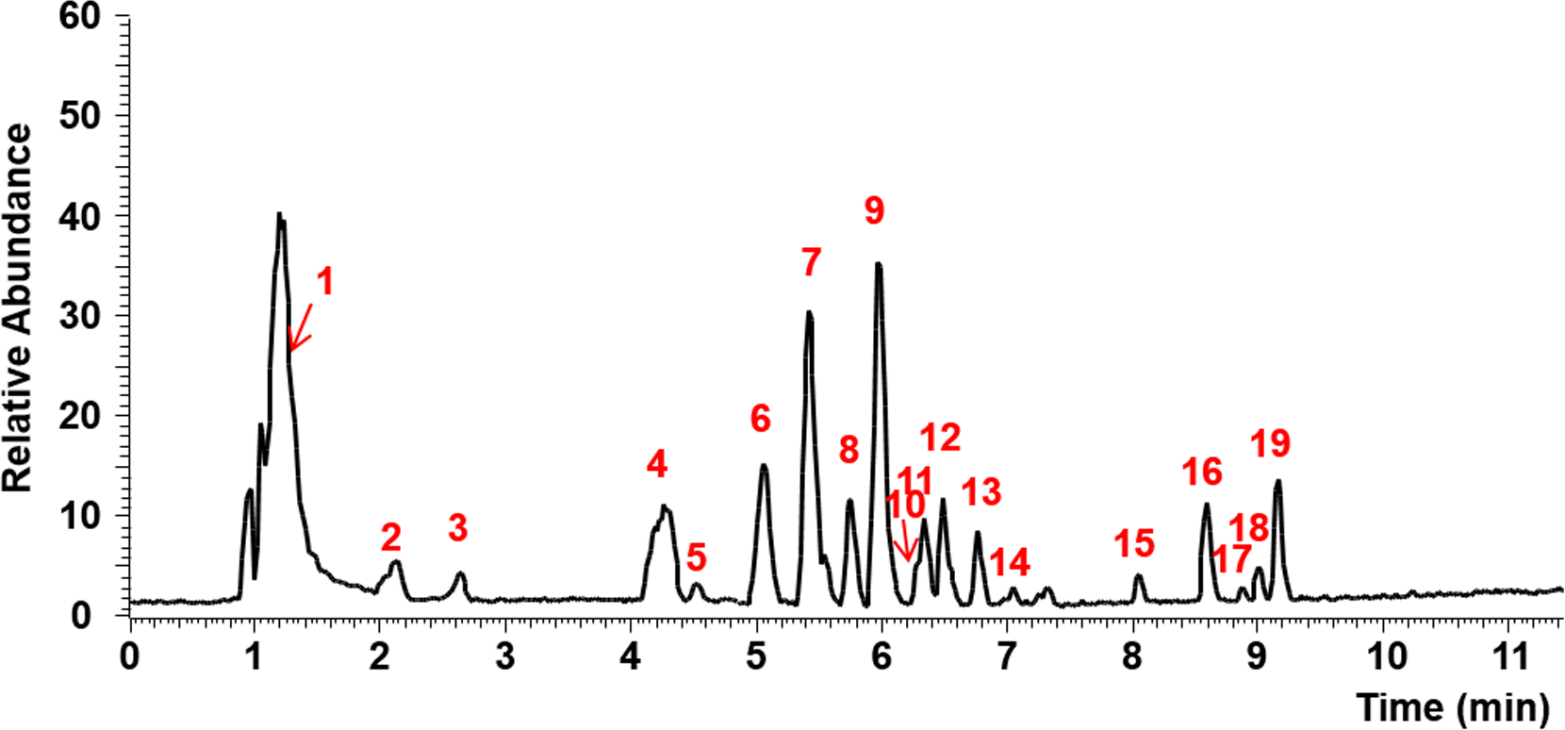
The total ion chromatogram of sample A in negative ion mode

The mass spectrometer was operated in full scan‐dd‐MS (Harper & Matsumoto, 2005) of positive and negative modes in a range of *m/z*: 70–1050. The resolution of MS data was set at 70,000, the acquired raw data were analyzed using Compound Discoverer 3.2 (Thermo Fisher Scientific) with the metabolite database (mzCloud, mzVault, Mass Lists, and ChemSpider). The bioactive components were analyzed by ultra‐performance liquid chromatography–electrospray–tandem mass spectrometry (UPLC–ESI–MS/MS) in both negative and positive modes. These chemical components in the sample A were either tentatively characterized or positively identified by comparing multistage and high‐resolution mass data and retention time with the corresponding data of commercial standards or earlier published data (Yan, Zhao, Chen, et al., 2018). The total ion current chromatogram of EUF is represented in Figure 1, and the compositions of sample A are summarized in Table 1.

**TABLE 1:** Nineteen compounds identified in sample A by the ultra‐performance liquid chromatography–electrospray–tandem mass spectrometry (UPLC–ESI–MS/MS) in negative ion mode

Peak no.	Compounds	Molecular formula	Retention time (min)	Precursor ion (m/z)	Main MS/MS ion fragments
1	Aucubin^b^	C_15_ H_22_ O_9_	1.27	[M+HCOO]^−^ 391.1239	183.0653, 155.0547, 139.0388
2	Asperulosidic acid^a^(Yan et al., 2018b)	C_18_ H_24_ O_12_	2.14	[M−H]^−^ 431.1185	251.0559, 165.0545, 89.0229
3	Chlorogenic acid^b^	C_16_ H_18_ O_9_	2.66	[M−H]^−^ 353.0871	191.0554, 85.0279
4	Geniposide^b^	C_17_ H_24_ O_10_	4.27	[M+HCOO]^−^ 433.1345	225.0763, 123.0438, 101.0229
5	Asperuloside^b^	C_18_ H_22_ O_11_	4.50	[M+HCOO]^−^ 459.1156	413.1085, 147.439, 119.0488
6	Quercetin‐3‐O‐β‐D‐glucopyranosyl‐(1 → 4)‐β‐D‐glucopyranoside^a^(Yan et al., 2018c)	C_27_ H_30_ O_17_	5.06	[M−H]^−^ 625.1393	463.0881,300.0270, 271.0254, 255.0300
7	Quercetin 3‐O‐sambubioside^a^(He et al., 2018)	C_26_ H_28_ O_16_	5.42	[M−H]^−^ 595.1295	300.0271, 271.0252, 255.0299
8	Rutin^b^	C_27_ H_30_ O_16_	5.75	[M−H]^−^ 609.1447	300.0270, 271.0252, 255.0300
9	Isoquercitrin^b^	C_21_ H_20_ O_12_	5.97	[M−H]^−^ 463.0890	300.0276, 271.0259, 255.0305
10	Kaempferol−3‐O‐rutinoside^a^(Yan et al., 2018b)	C_27_ H_30_ O_15_	6.25	[M−H]^−^ 593.1503	285.0407, 255.0301, 227.0345
11	Quercetin−3‐O‐(6”‐O‐acetyl)‐β‐D‐glucopyranoside^a^(Zhang et al., 2010)	C_23_ H_22_ O_13_	6.29	[M−H]^−^ 505.0986	300.0270, 271.0252, 255.0299
12	Astragalin^b^	C_21_ H_20_ O_11_	6.50	[M−H]^−^ 447.0935	284.0332, 255.0303, 227.0349
13	Kaempferol‐3‐O‐(6”‐O‐acetyl)‐β‐D‐glucopyranoside^a^(Yan et al., 2018c)	C_23_ H_22_ O_12_	6.80	[M−H]^−^ 489.1016	285.0405, 255.0302, 227.0347
14	Isochlorogenic acid C^a^(He et al., 2018)	C_25_ H_24_ O_12_	6.96	[M−H]^−^ 515.1182	353.0872, 191.0554
15	Eriodictyol^a^(Sonmezdag et al., 2018)	C_15_ H_12_ O_6_	8.06	[M−H]^−^ 287.0562	151.0024, 135.0438
16	Ethyl caffeate^a^(He et al., 2018)	C_11_ H_12_ O_4_	8.59	[M−H]^−^ 207.0656	179.0339, 161.0233, 135.0438
17	Apigenin^a^(Aghakhani et al., 2018)	C_15_ H_10_ O_5_	8.96	[M−H]^−^ 269.0460	225.1492, 151.0026, 117.0331
18	Naringenin^a^(Yan et al., 2018c)	C_15_ H_12_ O_5_	9.01	[M−H]^−^ 271.0618	177.0184, 151.0024, 119.0488
19	Kaempferol^b^	C_15_ H_10_ O_6_	9.13	[M−H]^−^ 285.0405	185.0595, 151.0024, 93.0329

^a^ Compared with metabolite database.

^b^ Compared with an authentic standard.

### 2.4 Dosage and treatment

Two hundred mice were randomized into 5 groups of 40 mice each. The groupings were as follows: control group, ethanol group (ethanol 6.0 g/kg), VE group (vitamin E 50 mg/kg +ethanol 6.0 g/kg), and sample A groups (100 mg/kg or 400 mg/kg +ethanol 6.0 g/kg). Eighty animals in sample A groups were orally administered with sample A at doses of 100 mg/kg and 400 mg/kg for 3 consecutive days, while the control group and the model group were given the same volume of 0.5% carboxymethylcellulose (CMC)–Na double‐distilled water for 3 consecutive days. Animals in the positive group were treated by gastric gavage with 50 mg/kg vitamin E (VE) for 3 consecutive days. Thirty minutes after treatment with vehicle, VE, or sample A, the sample A groups, positive group, and the ethanol‐only group were treated with an ip injection of ethanol at 6.0 g/kg (0.2 ml/10 g b.wt. (body weight)), and the control group was given an equal volume of saline using the same method. Ethanol was diluted to 30% (v/v) with saline for ip injection. All the animals were sacrificed at 4 h after the administration of ethanol.

### 2.5 Comet assay

DNA damage was assayed, according to our previously reported preceduries (Wu et al., 2018). In brief, the cerebellum and the cerebral cortex were minced, suspended at 1 ml/g in chilled homogenizing buffer, and then homogenized gently to obtain the precipitate for the analysis in comet assay. The comet assay was performed under alkaline conditions. Finally, the slide was stained with ethidium bromide (Amresco Inc., USA). One hundred cells were examined in each treatment group. The parameters were calculated using the automatic Comet Assay Software Project (CASP) image analysis system.

### 2.6 H&E dye method

Brain tissues in each group were used to prepare pathological sections, and hematoxylin–eosin (HE) staining, as we previously described (Wu et al., 2018).

### 2.7 MDA, NO, T‐SOD, 8‐OHdG, and GSH‐PX assays

Collected brain regions were suspended in chilled physiological saline, homogenized manually, and then centrifuged at 860 *g* for 10 min to obtain the supernatant. Centrifuge blood at 860 *g* for 15 min and gain the plasma. NO, MDA, T‐SOD, 8‐OHdG, and GSH‐PX were detected corresponding to the manufacturer's instructions.

### 2.8 Western blot analysis

The animal tissues were blended with precooled lysis buffer and homogenized with ultrasonic homogenizer. Then the homogenate was incubated in ice water bath for 30 min in the shaker. After being centrifuged at 13680 *g* for 10 min at 4°C, the supernatant was obtained as the total protein samples of tissues. The protein concentration was determined using a Bio‐Rad protein assay kit following the manufacturer's guide. Protein was separated on 12% sodium dodecyl sulfate–polyacrylamide gel (SDS‐PAGE) and transferred onto a polyvinylidene difluoride (PVDF) membrane. After blocking for 1 h, the membrane was incubated with primary antibodies (rabbit, 1:1000, Santa Cruz Biotechnology) at 4°C overnight, washed three times with Tris‐buffered saline with Tween‐20 (TBST), incubated with the horseradish peroxidise (HRP)‐labeled secondary antibody (antirabbit, 1:5000, Wuhan Google Biotechnology Co., Ltd) for1 h, and rewashed three times with TBST. The PVDF membrane was soaked with enhanced chemiluminescent (ECL) chromogenic reagent (chemiluminescent HRP substrate) for 5 min to blot. Imaging, fixing, and film processing were carried out in a dark room. The optical density (OD) of films was analyzed using the Alpha software to analyze and calculate the gray value in target bands, and was expressed as the ratio of gray level of target strips with β‐actin.

### 2.9 Molecular docking

The crystal structure of *Mus musculus* KEAP1 is retrieved from the Research Collaboratory for Structural Bioinformatics (RCSB) Protein Data Bank (PDB ID: 6QMC). The molecular docking was assessed using AutoDock Vina software. To prepare protein, water and the co‐crystallized ligand are deleted and hydrogens are added. The protonated state for each ionic residue is calculated and the conformation of the protein is optimized. The prepared protein is regarded as the receptor, and the binding site is defined by the PDB site record in the light of the location of ligand in 6QMC. The four main compounds are docked into the active site of Keap1 by using the Semi‐flexible Protein‐Ligand DOCKING method, respectively. The output protein–ligand complexes were produced with PyMol.

### 2.10 Statistical analysis

Results were expressed as mean ± SD. Data obtained were subjected to one‐way analysis of variance (ANOVA) test and complemented with Fisher's least significant difference (LSD) test. The statement of statistical significance was based on *p* < .05.

## 3 RESULTS

### 3.1 Sample analysis

The chemical components in sample A were tentatively or positively characterized by comparing the current mass data, including [M−H]^−^ m/z, MS/MS ion fragments, and retention time, with the corresponding data of commercial standards, metabolite database, or earlier published data (Yan, Zhao, Chen, et al., 2018). The ion current chromatogram of sample A is presented in Figure 1, and the detected chemical compositions were characterized and summarized in Table 1. A total of 19 components were detected, 12 of which were identified as flavonoids (**6**, **7**, **8**, **9**, **10**, **11**, **12**, **13**, **15**, **17**, **18**, and **19**), 3 of which were identified as phenolic acids (**3**, **14**, and **16**), and 4 of which were identified as iridoids (**1**, **2**, **4**, and **5**). It is the first time that compounds **15** and **17** have been described from *Eucommia* plant and compounds **6**, **7**, **10**, **11**, and **16** from *Eucommia ulmoides* Oliver male flower.

### 3.2 EUF decrease brain cell DNA damage induced by acute ethanol administration

**FIGURE 2:**
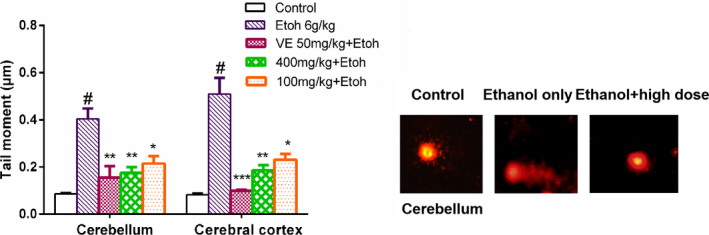
Effects of sample A against ethanol‐induced oxidative DNA damage in the cerebellum and the cerebral cortex of mice. Tail moments are shown as mean ± SD, ***p < .001 compared with the ethanol group, #*p* < .001 compared with the control group, *n* = 4

To analyze the potential protective effects of sample A following acute ethanol exposure in mice, DNA damage was investigated in isolated cells from mice cerebellum and cerebral cortex with or without pretreatment with sample A. VE treatment was chosen as a positive control (Guo et al., 2007). Treatment with sample A relieved DNA damage caused by ethanol in the mouse cerebellum and cerebral cortex, as shown by significantly lowered tail moment length after the ip administration of ethanol (Figure 2). Therefore, these results indicate that sample A at 100 and 400 mg/kg test doses affords protection against ethanol‐induced DNA damage, and the DNA protection of the high‐dose group was more significant than that of the low‐dose group.

### 3.3 Sample A decreases neuron cell damage

**FIGURE 3:**
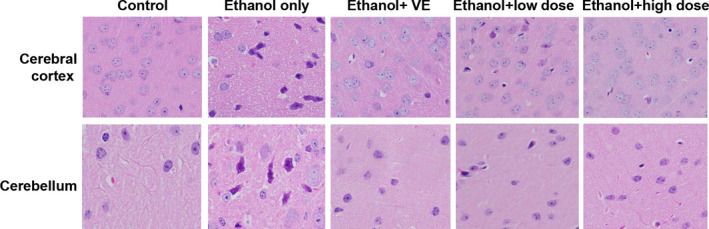
Action of sample A against ethanol‐induced histological changes in the cerebellum and cerebral cortex of mice (hematoxylin and eosin (H&E), ×400)

The histological slices from the control group had a normal number and arrangement of neurons in the cerebral cortex and in the cerebellum, with the nuclei also appearing morphologically normal in their size, shape, and arrangement (Figure 3). In mice administered ethanol alone, neurons were pyknotic with darkly stained and shrunken nuclei in hematoxylin and eosin (H&E)‐stained sections, indicating that alcohol caused serious DNA damage to the brain nerves of mice. Decreased neuronal damage was observed in mice brains by morphological observation under treatment with vitamin E (VE), prior to ethanol administration. Likewise, the cerebelli and cerebral cortices of mice administered sample A prior to ethanol administration also showed decreased presence of abnormal pyknotic neurons, with more neurons appearing normal in size, shape, and arrangement (Figure 3), suggesting that the neuronal damage was alleviated. The effect of the high‐dose group was markedly better than that of the low‐dose group by observing the cell morphology with fewer abnormal pyknotic neurons.

### 3.4 Decrease of NO, MDA, and 8‐OHdG concentrations in brain tissue and plasma by sample A

**FIGURE 4:**
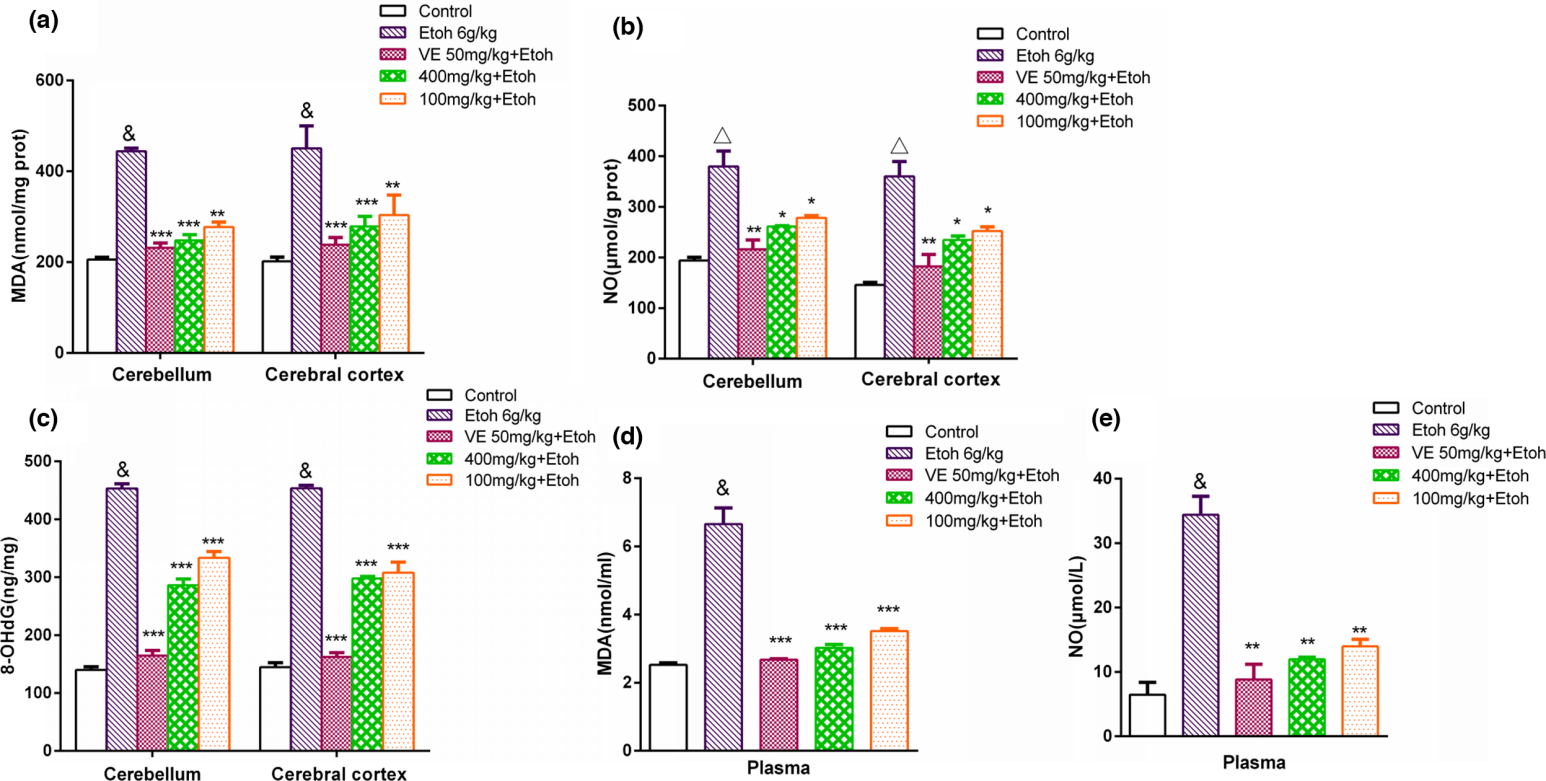
Actions of sample A on the levels of malondialdehyde (MDA), nitric oxide (NO), and 8‐hydroxy‐2′‐deoxyguanosine (8‐OHdG) in mice cerebellum, cerebral cortex, and plasma (mean ± SD, *n* = 4) are shown. (a) Levels of MDA in cerebellum and cerebral cortex; (b) levels of NO in cerebellum and cerebral cortex; (c) levels of 8‐OHdG in cerebellum and cerebral cortex; (d) levels of MDA in plasma; (e) levels of NO in plasma. &*p* < .001, △*p* < .01 compared with the control group; ***p < .001, **p < .01, *p < .05 compared with the ethanol group

As Figure 4 shows, a significant increase of NO, MDA, and 8‐OHdG levels was observed in the ethanol group compared with the control group, indicating that DNA damage is attributed to oxidative stress triggered by ethanol in brain tissue and blood. Pretreatment of EUF at 100 and 400 mg/kg obviously reduced the levels of NO, MDA, and 8‐OHdG in the mouse cerebellum, cerebral cortex, and plasma in comparison with those seen in the ethanol group. As expected, the relative potency for antioxidant activity was the high‐dose group >the low‐dose group.

### 3.5 Increase of T‐SOD and GSH‐PX activities in brain tissue and plasma by sample A

**FIGURE 5:**
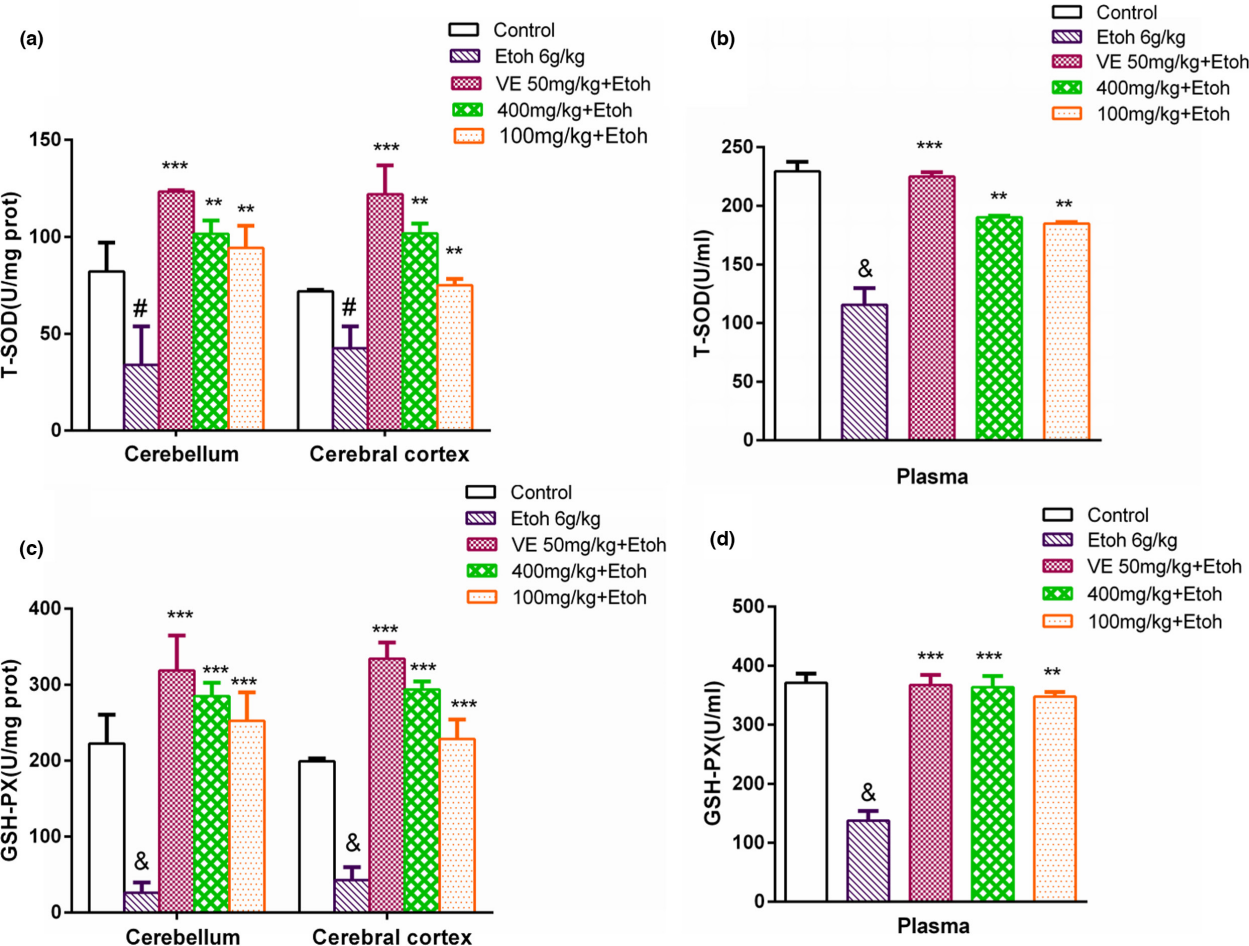
Actions of sample A on the total superoxide dismutase (T‐SOD) and glutathione peroxidase (GSH‐PX) activity in mice cerebellum, cerebral cortex, and plasma (mean ± SD, *n* = 4) are shown. (a) Activities of T‐SOD in cerebellum and cerebral cortex; (b) activities of T‐SOD in plasma; (c) activities of GSH‐PX in cerebellum and cerebral cortex; (d) activities of GSH‐PX in plasma. &*p* < .001, #*p* < .05 compared with the control group; ***p < .001, **p < .01 compared with the ethanol group

Seen from Figure 5, a marked decrease of T‐SOD and GSH‐PX activities was obtained in the ethanol group compared with the control group. T‐SOD and GSH‐PX activities in sample A groups become much higher than those in the ethanol group. The efficacy of high‐ dose group was superior to that of the low‐dose group.

### 3.6 Effects of sample A on the expression of Keap1 in Brain tissue after Acute Ethanol Administration

**FIGURE 6:**
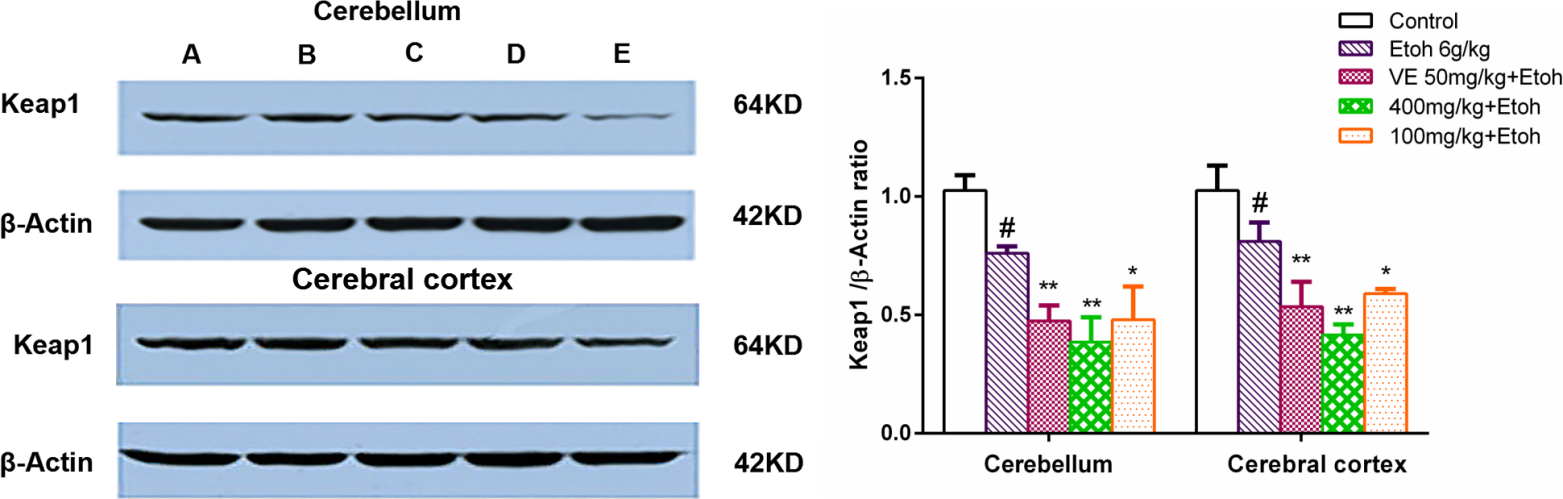
The expression of kelch‐like ECH‐associated protein‐1 (Keap1) in mice cerebellum and cerebral cortex. β‐Actin was used as housekeeping proteins. Data are presented as mean ± SD. #*p* < .05 versus the control group, *p < .05, **p < .01 versus the ethanol group. (Note: A. control group; B. ethanol group; C. vitamin E (VE) group; D. low‐dose group; E. high‐dose group)

To further investigate the possible role of sample A in protecting the brain from ethanol‐induced genotoxicity, the level of Keap1 involved in oxidative stress was detected in mouse cerebellum and cerebral cortex by western blotting. The protein expression of Keap1 was inhibited by ethanol stimulation, but the inhibitory effect was obviously enhanced by sample A treatment (Figure 6). These results further demonstrated that the protective mechanism of EUF on ethanol‐induced neuronal injury may be through downregulating the expression level of Keap1 protein to activate the Keap1–Nrf2 (nuclear factor‐erythroid 2‐related factor 2) signal pathway (Shen et al., 2018).

### 3.7 Molecular docking

**FIGURE 7:**
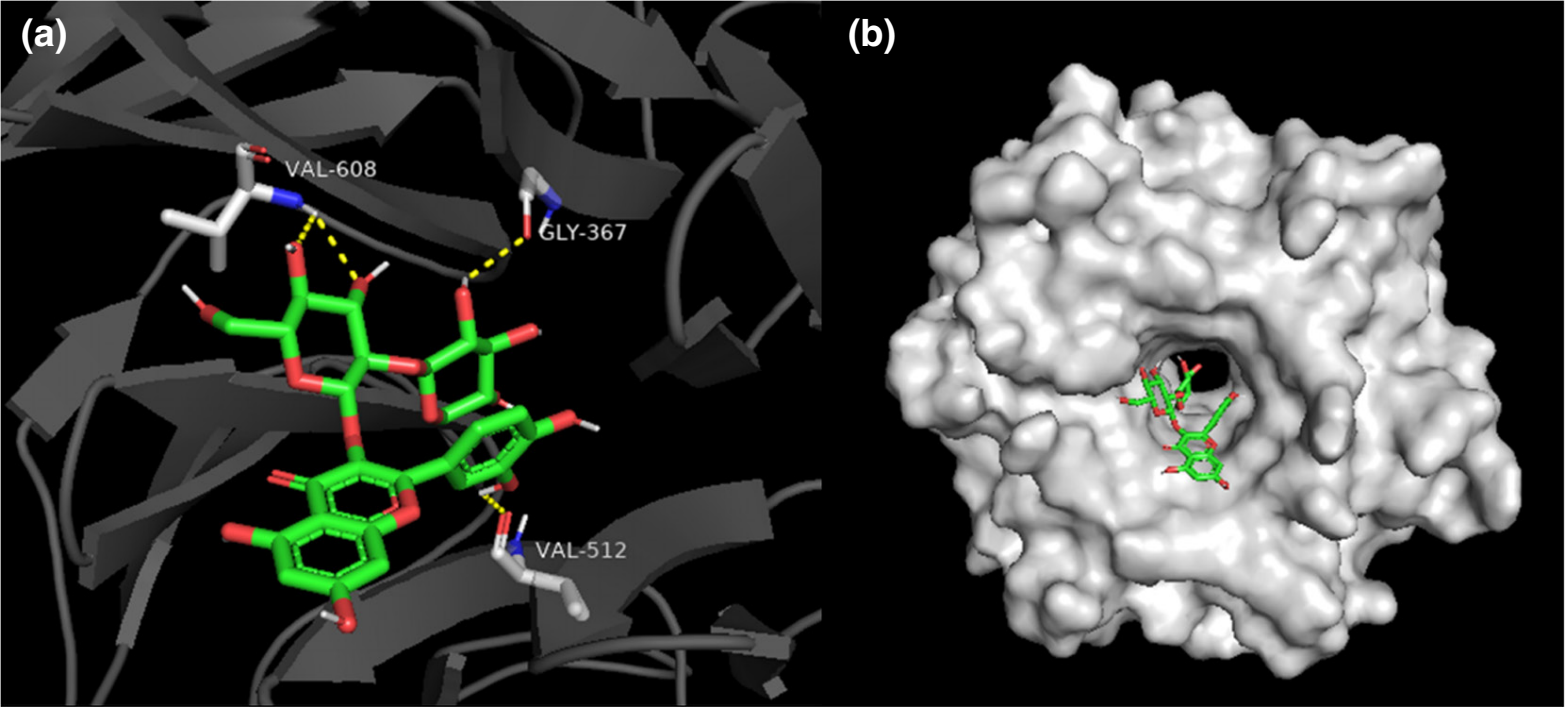
(a) Predicted binding mode of compound **7** and kelch‐like ECH‐associated protein‐1 (Keap1). Compound **7** is shown in green, and the key residues of Keap1 are shown in gray; (b) the panorama of interaction between compound **7** and Keap1. The dashed lines (yellow) represent hydrogen‐bonding interactions, and the Keap1 is shown as surface mode

In order to explore the neuroprotective mechanism of sample A, four main compounds, compounds **4**, **7**, **9**, and **16** with different structure types, are chosen to be docked into the binding site of Keap1by using the CDDOCK method, respectively. The binding energy is listed in Table 2. The lowest negative of interaction energy indicates that the interaction between ligand and protein is the strongest, and vice versa. Consequently, compound **7** was predicted to be the most likely inhibitor of Keap1 with interaction of −9.0 kcal/mol and accepting hydrogen bonds from Val608 Val512 Gly367. The three‐dimensional (3D) and the corresponding surface scheme for active sites of the complex of Keap1 and compound **7** are shown in Figure 7 and those of the complex between Keap1 and other compounds (**4**, **9**, and **16)** are, respectively, presented in Figures S1–S3. The simulation results revealed compounds **4**, **7**, **9**, and **16** actually interacted with the primary amino acid residues on the active site of Keap1 (Zhao et al., 2016).

**TABLE 2:** The interaction energy obtained by AUTODOCK for compounds **4**,**7**, **9**, and **16**

Compound	Docking interaction energy(kcal/mol)
**4**	−8.2
**7**	−9.0
**9**	−8.3
**16**	−6.7

## 4 DISCUSSION

*Eucommia ulmoides* Oliv., whose bark and leaf have been proven to possess medicinal activity (China Pharmacopoeia Committee, 2020), is one of the unique commercial tree species in China. Recent studies have demonstrated that the male flower of *E. ulmoides*, which is another major product of this tree species, is revealed to possess various physiological activities and functions, including hypolipidemic, antioxidant, antifatigue effects, etc. Among these, what is notable is the EUF’s well‐known protective activity against oxidative stress (Xing et al., 2019). In this work, effective fraction (sample A) of EUF was qualitatively analyzed by UPLC–ESI–MS/MS. Twelve flavonoids, 3 phenolic acids, and 4 iridoids were found in this sample, which demonstrated that sample A contained high concentrations of flavonoids with excellent antioxidative activity (Yao et al., 2009). In addition, there are some reports elsewhere about antioxidative effects of phenolic acids and iridoids existing in some foods and medicinal plants (Es‐Safi et al., 2007; Mudnic et al., 2010).

*Eucommia ulmoides* Oliv. leaf has been shown to be effective for the amelioration of ethanol‐induced oxidative DNA damage in the mouse brain, which may be attributed to the inhibition of ethanol‐induced oxidative stress (Fan et al., 2019). Recent research results have proved that some active compounds in EUF are almost the same as those in the leaves of *E*. *Ulmoides* Oliv., hence it was assumed that EUF would also own this protective property in brain tissue, which is worthwhile to investigate EUF for a possible role in attenuating ethanol‐induced genotoxicity. The results presented in Figure 2 showed that EUF treatment resulted in substantial reductions in comet tail moment spotted after ethanol treatment, and confirmed that EUF distinctly diminish the degree of DNA single‐strand breaks in mouse cerebellum and cerebral cortex after 6.0 g/kg ip ethanol was administered, which was further confirmed by morphological observation (Figure 3). Likewise, EUF showed strong DNA protective activity that was also observed in 8‐OHdG generation, a biomarker of oxidative DNA damage (Haghdoost et al., 2005). All the above results suggest that EUF could greatly alleviate ethanol‐induced oxidative DNA damage in mouse cerebellum and cerebral cortex.

It is widely known that acute alcohol intake brings about oxidative DNA damage in mammalian brain regions through oxidative stress. To ease this stress, the brain tissue stimulates endogenous antioxidant defense systems that involve enzymatic antioxidant mechanism mediated through SOD and GSH‐PX, and nonenzymatic antioxidant mechanism involving antioxidants such as vitamins and phenols (Maulik & Das, 2002; Seven et al., 2008). In this work, sample A was further explored into its action on the levels of NO, MDA, and 8‐OHdG, and SOD and GSH‐PX activities in mice cerebellum, cerebral cortex, and blood under oxidative stress. These results suggested sample A’s antioxidant effect by way of its promotion of SOD and GSH‐PX activity and inhibition of NO, MDA, and 8‐OHdG production. Seeing as the hydroxyl groups on benzene ring enable the compound to smoothly inactivate oxidative radicals (Sabuncuoglu et al., 2008), it was reckoned that flavonoids and phenolic acids in sample A would have the same antioxidant properties, because their structures all bear benzene rings with hydroxyl groups. In summary, sample A suppresses oxidative stress induced by ethanol via its potent antioxidant capacity (Yang et al., 2021; Zhang et al., 2022).

Generally, Keap1–Nrf2 is an important signaling pathway which is closely associated with oxidative stress. It regulates many downstream factors, such as SOD and GSH‐PX, which have many effects such as antioxidant stress, anti‐apoptosis, and so on (Lu et al., 2019). In this study, it was found that EUF could decrease the expression level of Keap1 protein and increase the ability of cells against oxidative stress to improve the protective function of the nervous system. Meanwhile, molecular docking analysis provided supportive data for the inhibition of Keap1 induced by compounds **4**, **7**, **9**, and **16**, allowing us to predict the binding site in the active site pocket (Zhao et al., 2018). Thus, we speculated that compounds **4**, **7**, **9**, and **16** may interact with Keap1 and occupy the Nrf2‐binding site on Keap1 protein, and disrupt the interaction of Nrf2 with the Keap1 Kelch domain, and finally induce the activation of Nrf2. These above results indicated that the Keap1–Nrf2 signaling pathway was activated to evoke an adaptive response to oxidative stress by regulating downstream antioxidant enzymes that serve to enhance cell survival. In a word, EUF may be a potential new natural product as a direct inhibitor of Keap1–Nrf2 interaction, which may ameliorate oxidative stress brain injury.

## 5 CONCLUSION

This study was first to identify 19 compounds for sample A using UPLC–ESI–MS. Compounds **15** and **17** have been extracted from the genus *Eucommia* and compounds **6**, **7**, **10**, **11**, and **16** from EUF for the first time. Sample A, the active fraction of EUF, was observed to alleviate ethanol‐induced oxidative DNA damage in mouse brain cells, possibly due to the activation of Keap1–Nrf2 pathway to inhibit oxidative stress. All this together makes EUF not only a promising ingredient for healthy beverages or other processed foods, but also a potential natural alternative or complement to the use of usual drugs for neuroprotection.

## ACKNOWLEGEMENTS

This work was supported by the National Science Foundation of China under grant number U2004129 and 82072726; the Key Scientific and Technological Project of Henan Province under grant number 212102311113; Scientific research project of Institute of traditional Chinese medicine of Henan University under grant number FX 1205A03 00001 and National Key Research and Develop Project under grant number 2017YFD0600702.

## CONFLICT OF INTEREST

The authors declare that they do not have any conflict of interest.

## Supporting information

Fig S1‐S3

## Data Availability

The datasets generated during and/or analyzed during the current study are available from the corresponding author upon reasonable request.
