# Supplementary material for: Protective effect of Eucommia ulmoides Oliver male flowers on ethanol‐induced DNA damage in mouse cerebellum and cerebral cortex
Source: Food Sci Nutr. 2022 Apr 22;10(8):2794–803. doi: 10.1002/fsn3.2882 (PMC9361448; doi:10.1002/fsn3.2882)
Supplement: Supplementary file 1 — Fig S1‐S3 [file FSN3-10-2794-s001.docx]

Supplementary Material


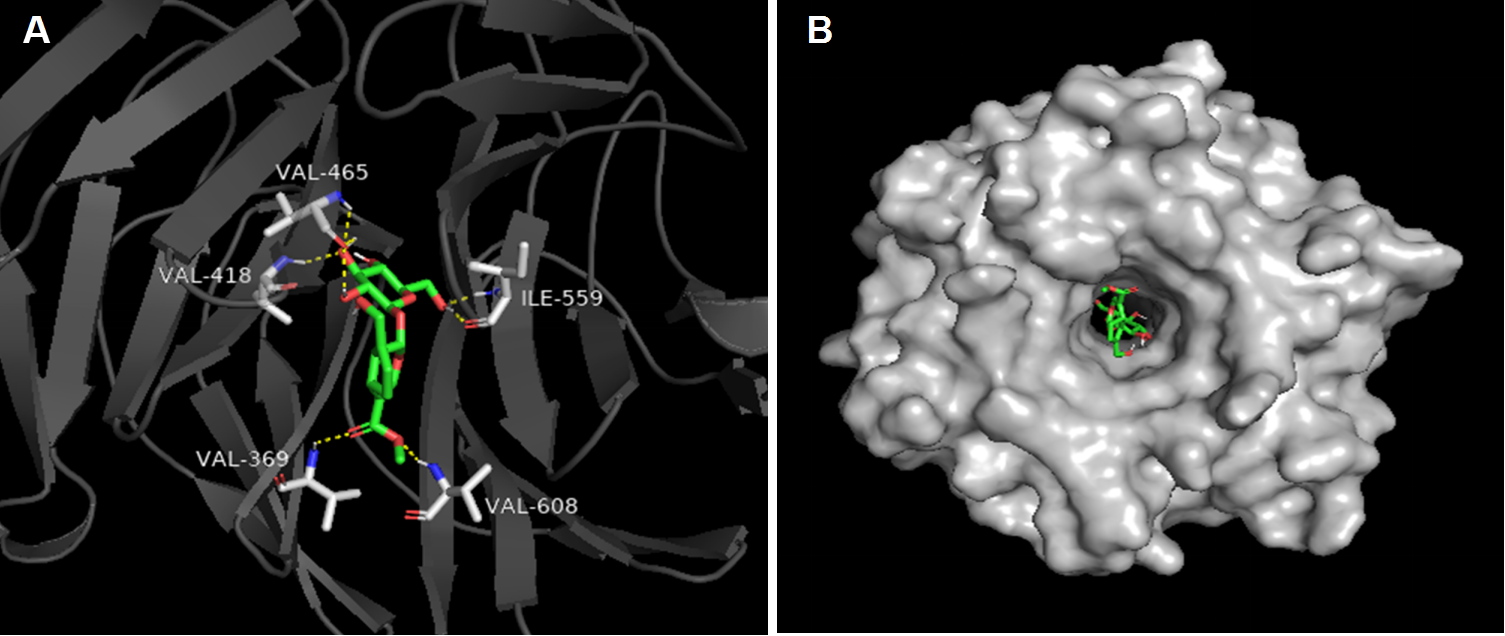


Figure S1 (A) Predicted binding mode of compound **4** and Keap1. Compound **4** is shown in green, and the key residues of Keap1 is shown in grey; (B) The panorama of interaction between compound **4** and Keap1. The dashed lines (yellow) represent hydrogen-bonding interactions, and the KEAP1 is shown as surface mode.


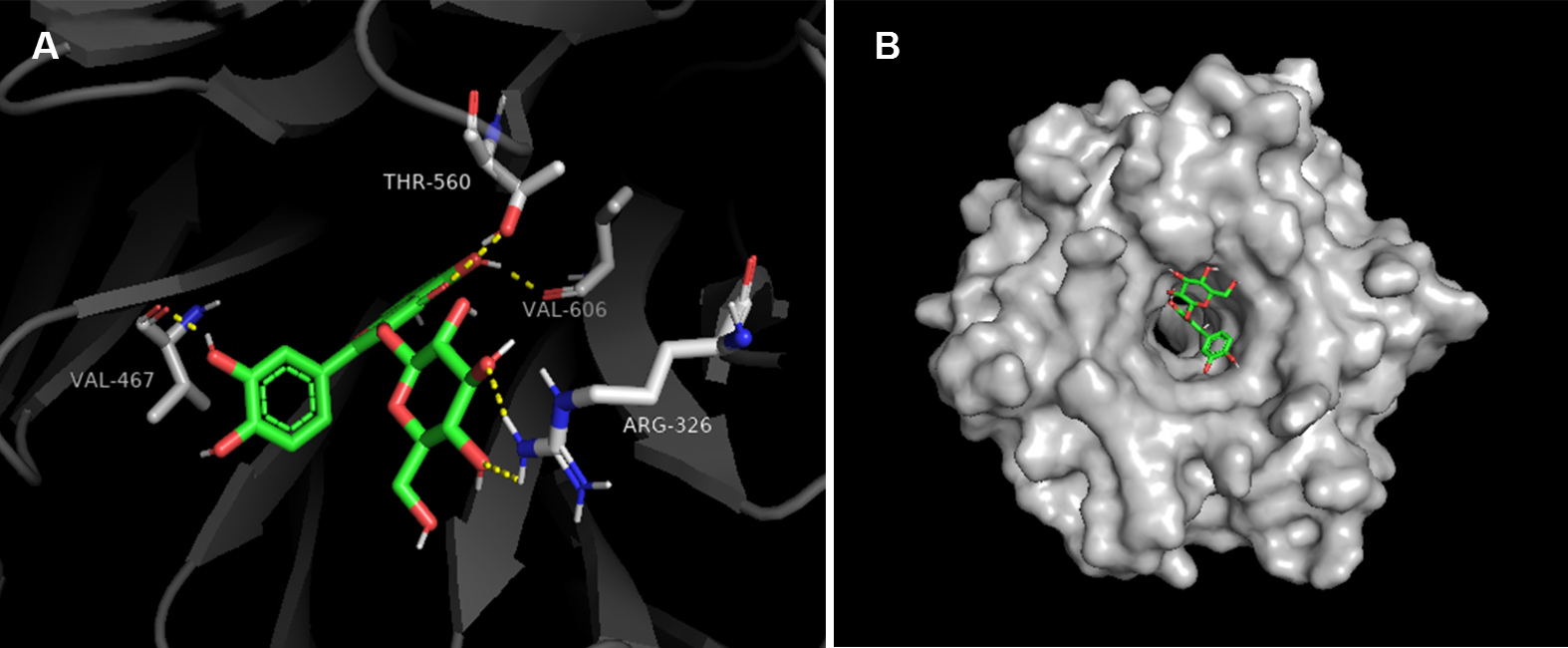


Figure S2 (A) Predicted binding mode of compound **9** and Keap1. Compound **9** is shown in green, and the key residues of Keap1 is shown in grey; (B) The panorama of interaction between compound **9** and Keap1. The dashed lines (yellow) represent hydrogen-bonding interactions, and the KEAP1 is shown as surface mode.


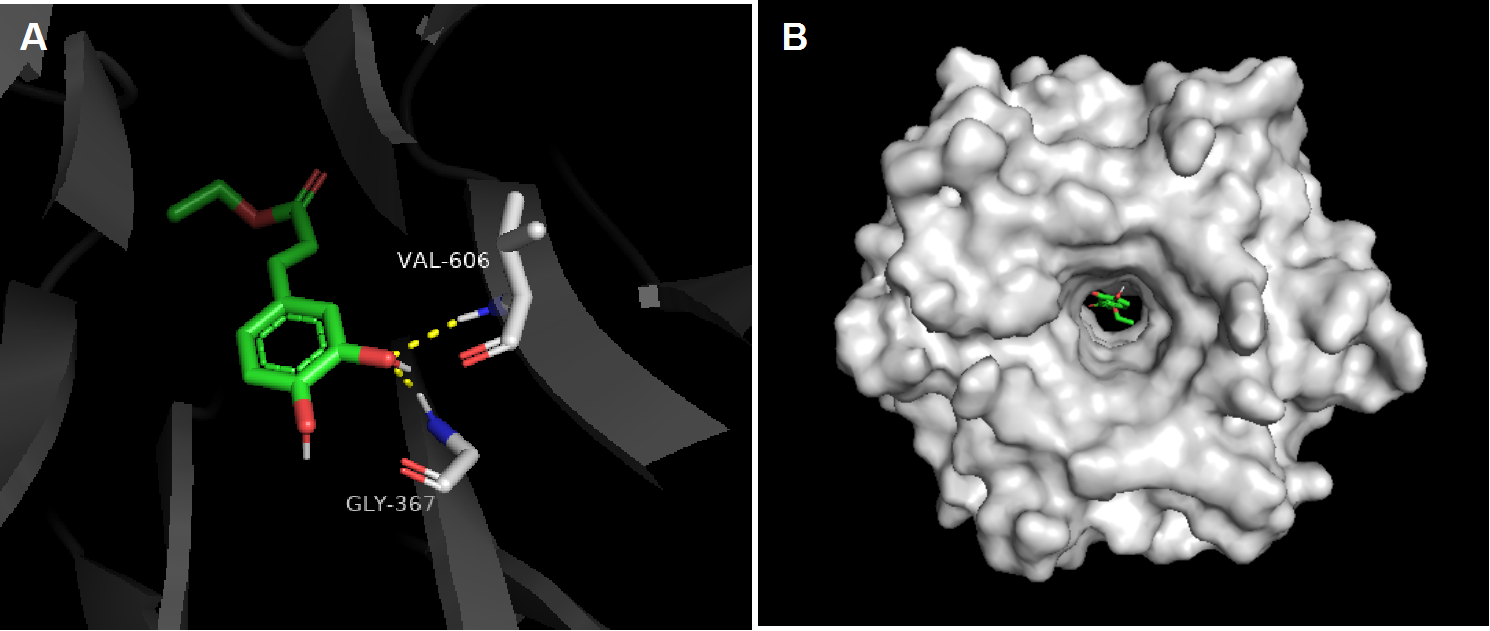


Figure S3 (A) Predicted binding mode of compound **16** and Keap1. Compound **16** is shown in green, and the key residues of Keap1 is shown in grey; (B) The panorama of interaction between compound **16** and Keap1. The dashed lines (yellow) represent hydrogen-bonding interactions, and the KEAP1 is shown as surface mode.
